# Impact of myocardial injury on regional left ventricular function in the course of acute myocarditis with preserved ejection fraction: insights from segmental feature tracking strain analysis using cine cardiac MRI

**DOI:** 10.1007/s10554-022-02601-3

**Published:** 2022-03-31

**Authors:** L. Weber, J. M. Sokolska, T. Nadarevic, M. Karolyi, B. Baessler, X. Fischer, M. Sokolski, J. von Spiczak, M. Polacin, I. Matziris, H. Alkadhi, M. Robert

**Affiliations:** 1 Institute of Diagnostic and Interventional Radiology, University Hospital Zurich, University of Zurich, Raemistrasse 100, 8091 Zurich, Switzerland; 2 Department of Radiology, Cantonal Hospital Winterthur, Winterthur, Switzerland; 3 Department of Cardiology, University Heart Center, University Hospital Zurich, University of Zurich, Zurich, Switzerland; 4 Department of Heart Diseases, Wroclaw Medical University, Wroclaw, Poland; 5 Department of Radiology, University Hospital Centre Rijeka, Rijeka, Croatia; 6 Department of Sport, Exercise and Health, University of Basel, Basel, Switzerland; 7 Institute for Biomedical Engineering, University and ETH Zurich, Zurich, Switzerland

**Keywords:** Cardiac magnetic resonance imaging, Late gadolinium enhancement, Myocarditis, Myocardial strain, Feature tracking strain analysis

## Abstract

The aim of this study was to provide insights into myocardial adaptation over time in myocyte injury caused by acute myocarditis with preserved ejection fraction. The effect of myocardial injury, as defined by the presence of late gadolinium enhancement (LGE), on the change of left ventricular (LV) segmental strain parameters was evaluated in a longitudinal analysis. Patients with a first episode of acute myocarditis were enrolled retrospectively. Peak radial (PRS), longitudinal (PLS) and circumferential (PCS) LV segmental strain values at baseline and at follow-up were computed using feature tracking cine cardiac magnetic resonance imaging. The change of segmental strain values in LGE positive (LGE+) and LGE negative (LGE−) segments was compared over a course of 89 ± 20 days. In 24 patients, 100 LGE+ segments and 284 LGE− segments were analysed. Between LGE+ and LGE− segments, significant differences were found for the change of segmental PCS (p < 0.001) and segmental PRS (p = 0.006). LGE + segments showed an increase in contractility, indicating recovery, and LGE− segments showed a decrease in contractility, indicating normalisation after a hypercontractile state or impairment of an initially normal contracting segment. No significant difference between LGE+ and LGE− segments was found for the change in segmental PLS. In the course of acute myocarditis with preserved ejection fraction, regional myocardial function adapts inversely in segments with and without LGE. As these effects seem to counterbalance each other, global functional parameters might be of limited use in monitoring functional recovery of these patients.

## Introduction

Acute myocarditis represents an inflammation of the myocardium, mostly caused by viral infection and frequently affecting young individuals. The clinical presentation is often unspecific, varying from subclinical disease and flu-like symptoms to a fulminant, infarct-like presentation with acute heart failure, arrhythmia, and sudden cardiac death [1, 2]. Myocarditis is frequently accompanied by electrocardiographic (ECG) alterations and elevated cardiac enzymes as a sign of myocardial ischemia, while left ventricular (LV) ejection fraction (EF) is often preserved [1, 3–5]. Whilst most patients recover completely, some develop myocardial dysfunction resulting in dilated cardiomyopathy. Although there is increasing research pertaining to the pathophysiology of myocarditis in regard to myocardial dysfunction, the effect of focal myocyte injury on regional function over the course of the disease remains poorly understood [6].

Cardiac magnetic resonance imaging (MRI) is the most sensitive imaging modality for the diagnosis and follow-up of patients with myocarditis, as it enables the combination of functional and morphological data in a multiparametric approach with high accuracy [7–11]. Moreover, cardiac MRI with late gadolinium enhancement (LGE) imaging can confirm myocyte injury and fibrotic transformation in the course of the disease. Subepicardial, multifocal patchy distribution of LGE in a non-ischemic pattern is characteristic of myocarditis [12, 13].

Myocardial dysfunction, as a typical characteristic of an inflammatory process, can be detected by visual assessment on cardiac MRI cine imaging; however, it is not always apparent in segments with LGE and is unspecific. Due to the frequent multifocal, patchy distribution of LGE, it was assumed that the surrounding myocardium might compensate the focal defect by increasing its own contractility, resulting in preserved regional and global function [10]. However, data supporting this hypothesis is lacking.

In current cardiac MRI practice, evaluation of myocardial contractility is generally performed by visual assessment on cine images, with inherent observer dependency [14]. Over the past years, feature tracking strain analysis using cine cardiac MRI was developed as a tool for the quantitative assessment of myocardial deformation, with the potential to detect subtle kinetic disorders and having the advantage of being less observer dependent [15–21].

The aim of this study was to provide insights into myocardial adaptation caused by myocyte injury over the course of acute myocarditis in a longitudinal analysis. The effect of myocardial injury, as defined by the presence of LGE, on change of left ventricular (LV) segmental strain parameters by feature tracking cardiac magnetic resonance (MRI) was evaluated.

## Materials and methods

### Study population

**Fig. 1:**
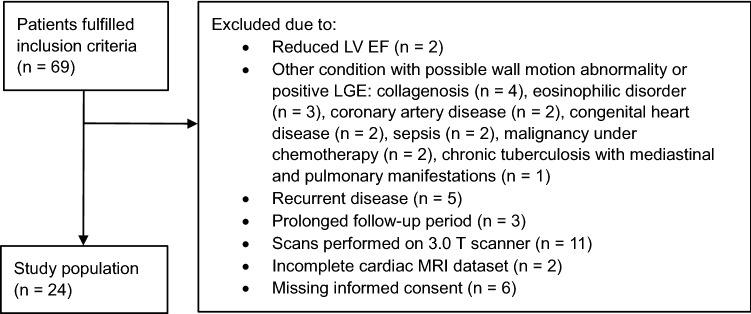
Study flow chart

We retrospectively enrolled consecutive patients who underwent baseline and follow-up cardiac MRI due to suspected myocarditis between January 2016 and December 2019 at our institution. Individuals treated as inpatients with their first clinical episode of myocarditis were included. Myocarditis was defined by the current European Society of Cardiology (ESC) guidelines [22]. Simultaneously the updated 2018 cardiac MRI criteria for non-ischemic myocardial inflammation had to be fulfilled, including positive late gadolinium enhancement imaging and presence of edema, either qualitatively on T2 black blood images or as global or regional increased T2 relaxation times on T2 mapping [10]. Patients with reduced LV EF or with any other medical condition possibly associated with a wall motion disorder or with presence of LGE were excluded from the study. Furthermore, outpatients and patients who did not undergo follow-up within six months after the first scan were excluded. Additionally, as segmental strain parameters are known to slightly depend on magnetic field strength, patients who underwent their follow-up cardiac MRI on a different scanner or both scans on the less often used 3.0T scanner were excluded [23, 24]. Finally, patients with incomplete cardiac MRI datasets and those who refused to provide written informed consent were excluded. Figure 1 displays the study flow chart.

Clinical data and laboratory values were collated from the patients’ electronic records. For the acute phase of myocarditis, peak inflammatory laboratory values and peak cardiac enzymes from serial blood sampling during hospitalisation were obtained. Clinical and laboratory follow-up was performed close to the follow-up MRI examination.

### Cardiac MR imaging

Cardiac MRI investigations were performed on a 1.5T system (Achieva, Philips Medical Systems, Best, The Netherlands) using a dedicated cardiac 5-channel phased array coil. For functional and feature tracking strain analysis, retrospectively triggered balanced steady-state free precession (b-SSFP) sequences in expiratory breath hold technique were acquired as a short axis stack covering the whole LV; repetition time 3.3 ms, echo time 1.6 ms, flip angle = 60°, spatial resolution = 8 × 1.5 × 1.5 mm^3^, in addition to three standard long axis views (2 chamber (CH), 3 CH and 4 CH). To visualise myocardial edema, fat saturated T2-weighted images were acquired in short axis orientation. Mapping sequences were only acquired in part of the population, which is why the data is not displayed under results. LGE imaging was performed 10 min after administration of 0.2 mmol/kg contrast agent (Gadovist, Bayer Healthcare, Germany) using a gradient-spoiled turbo fast-field-echo sequence with a non-selective 180° inversion pre-pulse in end diastole. The optimal inversion time was obtained from a Look Locker sequence.

### MR imaging analysis

Cardiac MRI data was analysed on a commercially available post-processing software (Intellispace Portal, Version 8, Philips Healthcare). Volumetric analysis was performed according to the current guidelines of the Society of Cardiovascular Magnetic Resonance (SCMR). LV and right ventricular (RV) volumes, as well as LV myocardial mass were indexed to the calculated body surface area [25]. Global myocardial fibrosis was quantified using the full width at half maximum (FWHM) method [26]. Distribution of LGE was reported as subepicardial, intramural or subendocardial, using the 16-segment model of the American Heart Association (AHA) [27].

A dedicated software (Segment CMR, Version 3, Medviso, Lund, Sweden) was used for feature tracking strain analysis of cardiac MRI cine images, known for its excellent intra- and interobserver reproducibility [18, 28]. The software computes myocardial strain curves from inter-frame deformation fields derived by non-rigid image registration [29]. To ensure comparability, the same reader, who followed a clear protocol, performed strain analysis. Circumferential and radial strain was assessed using basal, midventricular, and apical short axis images. The basal slice was defined as the slice immediately basal to the tips of the papillary muscles, the apical slice as the first slice on which no definite papillary muscle could be delineated any more.

Longitudinal strain was assessed using 2-chamber, 3-chamber, and 4-chamber long axis slides. Endo- and epicardial contours were drawn manually, and propagated automatically throughout the cardiac cycle. The quality of propagation was checked visually, and if necessary, manual contouring was adapted and propagation was repeated. Segmental strain parameters were derived according to the 16-segment model of the AHA.

**Fig. 2:**
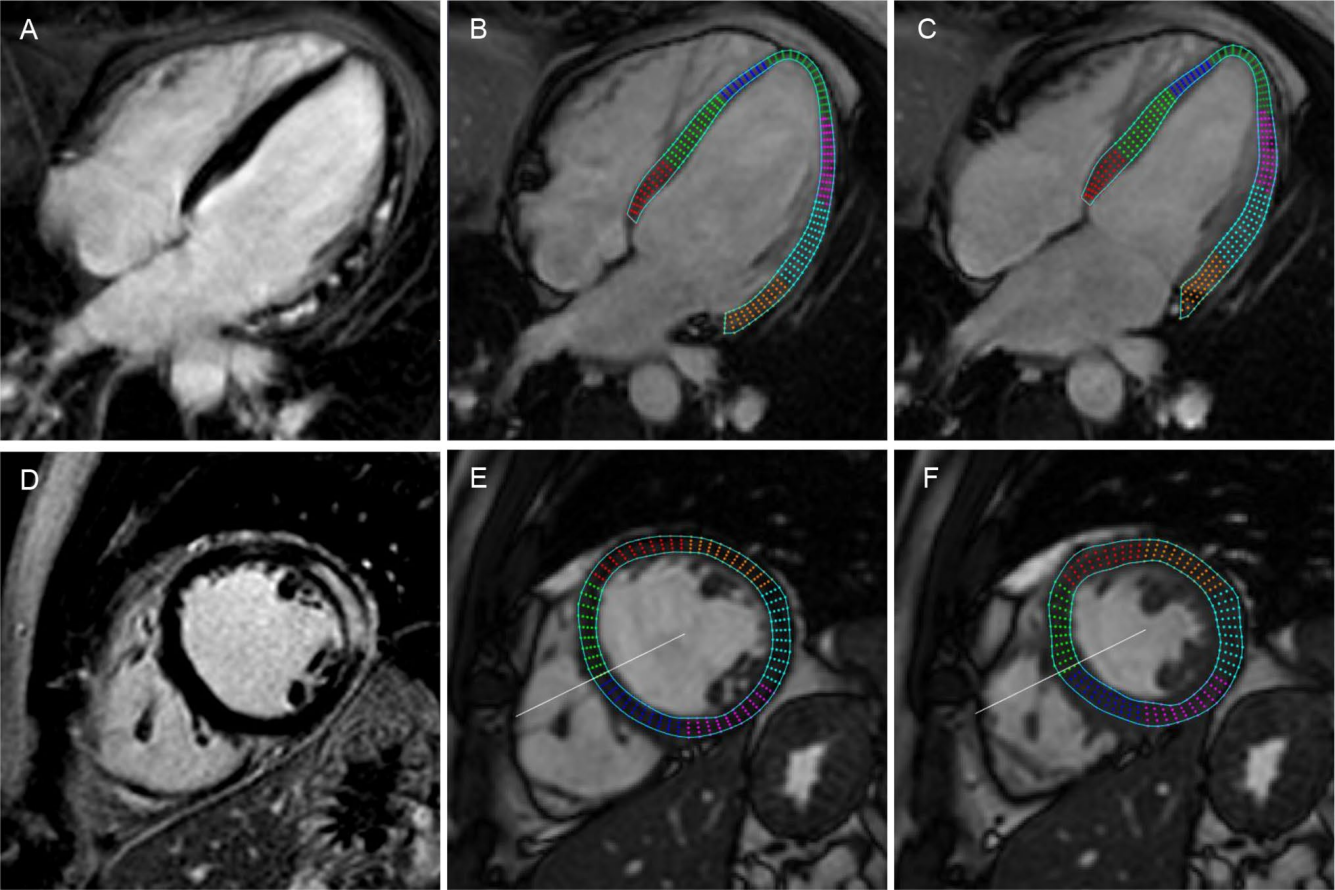
Example of Late Gadolinium Enhancement imaging and feature tracking strain analysis by cardiac magnetic resonance in a patient with acute myocarditis. A representative 4 chamber (**A**) and a short axis midventricular LGE image (**D**) show extensive involvement of the lateral left ventricular wall. Longitudinal strain analysis is performed in the long axis view as represented by an enddiastolic (**B**) and endsystolic 4 chamber slice (**C**). Circumferential and radial strain analysis is assessed using short axis slices as represented by an enddiastolic (**E**) and endsystolic (**F**) midventricular slice

Late gadolinium enhancement imaging and strain analysis are illustrated in Fig. 2.

### Statistics analysis

Population characteristics are presented using means and standard deviations (SD) for continuous variables as well as counts and percentages for categorical variables. Descriptive statistics (histograms) were applied to test the normality of data. The Student’s t test for paired samples was used to compare clinical characteristics between baseline and follow-up.

Strain values of specific segments are displayed as means and standard deviations of their absolute values. Since segmental strain values are known to show different distributions in different segments [18, 29], these values were z-standardised in order to summarise LGE+ and LGE− segments for comparison at baseline.

Change in segmental strain values from baseline to follow-up was computed by subtracting baseline values from follow-up values. These changes were expressed as their standardised mean difference (SMD) with 95% confidence intervals (CI). The strain values of all LGE+ and LGE− segments were compared using a Student’s t test for unpaired samples.

Statistical significance was set as a two-sided p-value of < 0.05. All statistical analyses were computed using the STATA version 15.0 (StataCorp, College Station, TX, USA).

## Results

### Patient characteristics

We included 24 patients (83% male, 17% female, mean age 28.9 ± 10.7 years) with acute myocarditis. Baseline demographic and clinical characteristics of the study population are displayed in Table 1. The average length of stay in our hospital was 4.5 ± 2.3 days. On average, the baseline cardiac MRI scan was conducted 4.6 ± 3.1 days and the follow-up cardiac MRI scan 93.6 ± 19.9 days after admission. Table 2 shows clinical characteristics, as well as laboratory and ECG parameters for each time point.

**Table 1:** Characteristics of myocarditis patients at baseline

Parameter	n = 24
Demographics, basic vital signs and body indices	
Gender (male/female)	20 (83%)/4 (17%)
Age (years)	28.9 ± 10.7
Height (cm)	175.9 ± 8.1
Weight (kg)	77.2 ± 10.9
BMI (kg/m^2^)	25.0 ± 3.3
BSA (m^2^)	1.9 ± 0.2
Heart rate (bpm)	81.3 ± 23.2
Blood pressure systolic/diastolic (mmHg)	126.0 ± 13.7 / 77.2 ± 12.1
ECG Criteria consistent with myocarditis	
ST-segment elevation > 0.1 mV in at least one lead	9 (38%)
ST-segment depression > 0.1 mV in at least one lead	3 (13%)
T-inversion in at least one lead	11 (46%)
Clinical symptoms consistent with myocarditis	
Acute chest pain	21 (88%)
Dyspnoea	4 (17%)
Palpitations/arrhythmia symptoms/syncope	5 (21%)
History of prior infection	
Flue like/respiratory	15 (63%)
Gastrointestinal	5 (21%)
Other^a^	2 (8%)
Cardiovascular risk factors	
Arterial hypertension	none
Diabetes	1 (4%)
Dyslipidaemia	none
Smoking	14 (58%)
Active	11 (46%)
Exclusion of coronary artery disease	
Invasive coronary angiography	9 (38%)
Cardiac computed coronary angiography	7 (29%)
Clinically (age < 30 years)	6 (25%)

Data are means ± standard deviations (SD) or number of patients with percentages in parentheses

*bpm* Beats per minute, *BMI* body mass index, *BSA* body surface area (Mosteller), *ecg* electrocardiogram

^a^Dental extraction/exanthema

**Table 2:** Clinical and cardiac MRI characteristics at baseline and follow-up

Parameter	Baseline	Follow-up	p-value
Laboratory criteria			
hs troponin max (ng/l)	898.2 ± 733.6	6.5 ± 4.0 (n = 20)	< 0.001
CK max (U/I)	380.5 ± 289.5	142.1 ± 116.6 (n = 18)	0.004
Myoglobine max (µg/l)	85.4 ± 106.3	27.8 ± 7.1 (n = 14)	0.027
NT pro BNP max (ng = l)	795.9 ± 1640.5	27.0 ± 23.9 (n = 21)	0.044
CRP max (ml/l)	40.4 ± 40.3	1.3 ± 1.4 (n = 22)	< 0.001
White blood cell count max (G/l)	9.8 ± 2.9	6.6 ± 1.3 (n = 22)	< 0.001
Cardiac MRI			
LVEDVi (ml/m^2^)	90.6 ± 13.3	87.2 ± 11.7	0.034
LVESVi (ml/m^2^)	40.0 ± 8.2	38.3 ± 8.0	0.155
LVEF (%)	56.0 ± 4.5	56.4 ± 4.7	0.691
LVMassi (g/m^2^)	52.9 ± 11.6	47.5 ± 8.9	0.001
RVEDVi (ml/m^2^)	86.9 ± 14.1	85.4 ± 11.2	0.302
RVESVi (ml/m^2^)	36.4 ± 8.0	37.5 ± 7.7	0.368
RVEF	58.4 ± 4.6	56.4 ± 5.3	0.078
LGE quantification (FWHM)	6.5 ± 3.8	4.5 ± 4.7	0.023
Global Peak Strain Parameters			
GLS	− 13.0 ± 2.0	− 13.3 ± 1.6	0.337
GRS	34.9 ± 7.9	33.0 ± 9.5	0.223
GCS	− 17.6 ± 2.6	− 17.2 ± 4.3	0.625

Data are means ± standard deviations (SD)

Unless otherwise specified: n = 24

*max* maximum during hospitalisation, *hs troponine* high sensitive troponine, *CK* creatine kinase, *NT pro BNP* N-terminal pro b-type natriuretic peptide, *CRP* C-reactive protein, *LVEDVi* left ventricular end diastolic volume / body surface area (BSA), *LVESVi* left ventricular end systolic volume (BSA), *LVEF* left ventricular ejection fraction, *LVMassi* left ventricular myocardial mass (BSA), *RVEDVi* right ventricular end diastolic volume (BSA), *RVESVi* right ventricular end systolic volume (BSA), *RVEF* right ventricular ejection fraction, *LGE* late gadolinium enhancement, *FWHM* full width half maximum, *GLS* global peak longitudinal strain, *GRS* global peak radial strain, *GCS* global peak circumferential strain

### Late gadolinium enhancement

Consistent with the study inclusion criteria, LGE was present in all patients. At baseline, a total of 100 LGE+ and 284 LGE− segments were detected. This corresponds to an average of 4.2 ± 2.8 affected segments per patient. The segmental distribution of LGE is listed in Table 3. LGE was most frequently present in the inferolateral basal (n = 18, 72%), inferolateral midventricular (n = 15, 60%) and anterolateral midventricular (n = 12, 48%) segments. Antero- and inferoseptal basal as well as anteroseptal midventricular segments were not affected in any of the study patients. Overall, the pattern of LGE+ segments was predominantly subepicardial (93%).

**Table 3:** Segmental strain values (%) in LGE+ and LGE− segments

	N of LGE+ at baseline	Peak circumferential strain				Peak radial strain				Peak longitudinal strain			
		Baseline		Follow-up		Baseline		Follow-up		Baseline		Follow-up	
		LGE+	LGE−	LGE+	LGE−	LGE+	LGE−	LGE+	LGE−	LGE+	LGE−	LGE+	LGE−
Basal segments													
Anterior	2 (8%)	− 16 ± 1	− 18 ± 5	− 13 ± 1	− 16 ± 5	18 ± 1	39 ± 11	21 ± 2	39 ± 13	− 13 ± 9	− 13 ± 8	− 15 ± 5	− 15 ± 5
Anteroseptal	0 (0%)		− 19 ± 4		− 17 ± 4		21 ± 13		25 ± 12		− 17 ± 7		− 18 ± 6
Inferoseptal	0 (0%)		− 18 ± 4		− 18 ± 5		16 ± 11		17 ± 11		− 22 ± 6		− 21 ± 6
Inferior	10 (40%)	− 6 ± 3	− 9 ± 7	− 10 ± 4	− 9 ± 5	39 ± 16	32 ± 17	42 ± 11	32 ± 15	− 31 ± 7	− 24 ± 7	− 30 ± 6	− 27 ± 9
Inferolateral	18 (72%)	− 13 ± 3	− 14 ± 5	− 14 ± 3	− 16 ± 5	27 ± 12	35 ± 24	32 ± 11	37 ± 16	− 27 ± 8	− 26 ± 13	− 32 ± 9	− 32 ± 10
Anterolateral	9 (36%)	− 16 ± 3	− 18 ± 6	− 15 ± 4	− 17 ± 5	29 ± 10	40 ± 17	30 ± 7	32 ± 11	− 14 ± 9	− 17 ± 7	− 20 ± 8	− 18 ± 8
Midventricular segments													
Anterior	3 (12%)	− 17 ± 6	− 21 ± 4	− 14 ± 4	− 18 ± 5	42 ± 18	45 ± 11	43 ± 9	38 ± 11	− 13 ± 3	− 16 ± 5	− 14 ± 7	− 16 ± 4
Anteroseptal	1 (4%)	− 19 ± 0	− 18 ± 3	− 16 ± 0	− 16 ± 6	41 ± 0	32 ± 11	26 ± 0	28 ± 12	− 17 ± 0	− 13 ± 5	− 19 ± 0	− 11 ± 4
Inferoseptal	0 (0%)		− 18 ± 3		− 18 ± 4		35 ± 9		32 ± 12		− 9 ± 6		− 8 ± 4
Inferior	4 (16%)	− 8 ± 2	− 10 ± 4	− 10 ± 6	− 12 ± 3	27 ± 8	36 ± 9	39 ± 6	36 ± 12	− 7 ± 5	− 10 ± 5	− 6 ± 6	− 11 ± 3
Inferolateral	15 (60%)	− 12 ± 5	− 16 ± 6	− 16 ± 3	− 11 ± 8	38 ± 13	39 ± 10	46 ± 9	38 ± 14	− 9 ± 4	− 12 ± 5	− 10 ± 4	− 10 ± 6
Anterolateral	12 (48%)	− 10 ± 4	− 13 ± 3	− 12 ± 5	− 14 ± 4	42 ± 9	42 ± 11	39 ± 15	40 ± 11	− 10 ± 6	− 8 ± 6	− 8 ± 3	− 7 ± 6
Apical segments													
Anterior	9 (36%)	− 23 ± 6	− 22 ± 5	− 24 ± 7	− 18 ± 7	43 ± 9	37 ± 15	40 ± 13	28 ± 15	− 12 ± 5	− 11 ± 2	− 12 ± 4	− 11 ± 3
Anteroseptal	2 (8%)	− 24 ± 4	− 27 ± 5	− 24 ± 2	− 25 ± 8	27 ± 2	27 ± 13	17 ± 8	24 ± 16	− 8 ± 3	− 11 ± 3	− 1 ± 12	− 11 ± 4
Inferoseptal	4 (16%)	− 19 ± 6	− 22 ± 5	− 27 ± 5	− 22 ± 7	42 ± 6	39 ± 11	33 ± 13	34 ± 14	− 7 ± 3	− 8 ± 5	− 8 ± 2	− 8 ± 4
Inferior	11 (44%)	− 17 ± 7	− 18 ± 4	− 20 ± 7	− 15 ± 9	49 ± 11	43 ± 16	46 ± 15	38 ± 17	− 8 ± 4	− 9 ± 3	− 9 ± 3	− 8 ± 3

At follow-up, the number of LGE+ segments had decreased to an average of 2.8 ± 2.5 per patient with no new segments detected. The pattern of affected segments remained similar, with inferolateral basal and midventricular segments being most frequently affected.

The summarised amount of LGE, calculated by the FWHM method, was inter-individually highly variable (mean at baseline = 6.5 ± 3.9%), and declined significantly from baseline to follow-up (p = 0.023).

### Global functional parameters

LV ejection fraction as well as global strain values are displayed in Table 2. Patients showed an average LV EF of 56 ± 4.5%. There was no significant difference of global peak longitudinal strain (PLS; p = 0.337), global peak radial strain (PRS; p = 0.223), or global peak circumferential strain (PCS; p = 0.625) values between baseline and follow-up assessment.

### Baseline regional functional parameters

Absolute values of segmental strains at baseline and at follow-up are displayed in Table 3. For segmental PCS at baseline, the sum of all z-standardised values of LGE+ segments showed lower strain values compared to LGE− segments (p < 0.001). For PRS (p = 0.163) and PLS (p = 0.701) at baseline, segmental strain values in LGE+ and LGE− segments did not show a significant difference.

### Change in segmental strain values

**Fig. 3:**
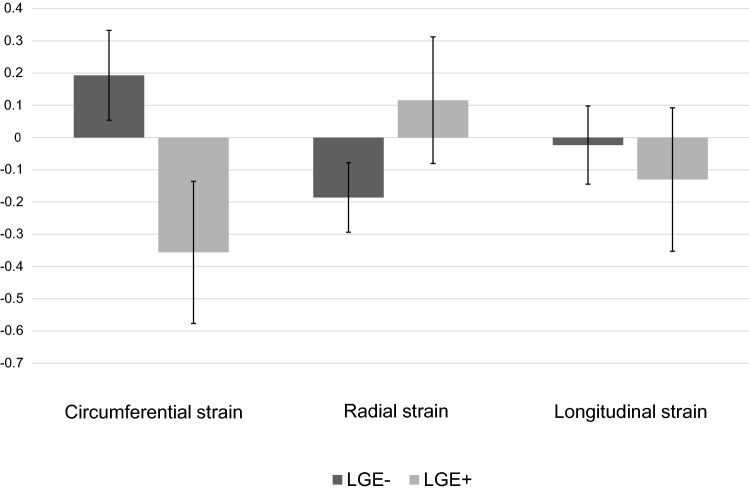
Change in segmental strain values from baseline to follow-up. Bars indicate the standardised mean difference (SMD) and error bars their 95% confidence interval. As peak circumferential strain and peak longitudinal strain are expressed as negative percentages, negative SMD indicates increase, positive SMD indicates decrease in contractility. For peak radial strain the inverse is the case. *LGE*+ Segments with late gadolinium enhancement, *LGE− *Segments without late gadolinium enhancement

Changes in peak segmental strain values from baseline to follow-up for LGE+ and LGE− segments are displayed in Fig. 3. Standardised mean difference of segmental strain values in LGE+ and LGE− segments differed significantly for PCS (p < 0.001) and PRS (p = 0.006), but showed no difference for PLS (p = 0.387). PCS showed an increase of contractility in LGE+ segments (standardised mean difference (SMD) = − 0.36; 95% CI − 0.58 to − 0.14) and a decrease in LGE− segments (SMD = 0.19; 95% CI 0.05 to 0.33). Likewise, PRS of LGE + segments showed a trend towards higher contractility (SMD = − 0.12; 95% CI − 0.08 to 0.31), whereas LGE− segments tended towards lower contractility (SMD = − 0.19; 95% CI = − 0.29 to − 0.08). Concerning PLS, we observed no significant change over time in LGE+ (SMD = -0.13; 95% CI -0.35 to 0.09) and LGE− segments (SMD = − 0.02; 95% CI − 0.14 to 0.10).

## Discussion

To the best of our knowledge, this is the first study focusing on the regional effect of myocyte injury on myocardial function during the course of acute myocarditis with preserved ejection fraction, using feature tracking strain analysis. Thus, the study provides insights into the potential mechanisms of myocardial dysfunction and its compensatory mechanisms.

Feature tracking strain analysis was able to depict differences in LGE+ segments compared to LGE− segments for PCS and PRS. On the one hand, LGE+ segments showed a significant improvement in PCS and a trend towards improvement in PRS, indicating recovery. On the other hand, LGE− segments showed a reduction in PCS and PRS indicating normalisation from an initially hypercontractile state or impairment of an initially normal contracting segment. For PLS, no significant change could be observed.

Our data supports the hypothesis that myocyte injury in the acute stage of myocarditis leads to regional myocardial dysfunction and recovers over time. Adjacent myocardium, which was affected to a lesser extent or completely unaffected, seems to react with an initial hypercontractility to compensate and seems to normalise once recovery sets in. An alternative explanation for the inverse reaction of initially unaffected segments would be the development of a scar, however in the absence of progression of LGE, this appears less likely.

There is limited data in the literature on the correlation between presence of LGE and segmental myocardial strain parameters. However, as a proof of concept for this study, Tahir et al. [18] found reduced radial segmental strain values in LGE+ segments or in directly adjacent segments in a cohort of competitive triathletes, using the same feature tracking analysis software. In a small study of 10 children with acute myocarditis, Uppu et al. [30] found a moderate correlation between segmental PLS values derived by echocardiographic two-dimensional speckle-tracking strain analysis and presence of LGE in cardiac MRI. Meindl et al. [31] similarly found reduced segmental longitudinal strain in 2D echocardiography in the inferior and inferolateral segments, which in their study as well were often affected by LGE.

Regarding the course of global peak strain values in acute myocarditis, Luetkens et al. [32] found significant improvement of global LV strain values from baseline to follow-up. Conversely, our study showed no significant change in global peak systolic strain values. However, these results might not be directly comparable due to differences in study populations, as there were patients with reduced ejection fraction and being of older age included in Luetkens et al. [32].

The present data shows that compensatory mechanisms of the LV myocardium occurs over the course of acute myocarditis in patients with preserved ejection fraction. At the segmental level, LGE + and LGE− segments adapt inversely in PCS and PRS. Meanwhile global strain parameters remain unchanged over time, underlining the limited utility of global function parameters to track functional recovery over the course of acute myocarditis in patients with preserved ejection fraction.

PLS shows no significant changes at either segmental or global level. Whether the lack of change is due to a limitation of the software used in the study, or a lack of remodelling possibilities cannot be determined from our data.

In the acute phase of myocarditis, reduced global strain values have been observed in several studies [16, 17, 21, 30–32]. According to our research topic, which should cover the temporal course of myocarditis, we did not correlate the results using an additional healthy control group. However, the values of the control group of Tahir et al. [18] should be reasonably comparable, since a similar scanner was used and the strain analysis was performed with the same software. In our myocarditis population, global circumferential strain values are similar to the control cohort evaluated by Tahir et al. [18], whereas global longitudinal and radial strain values are restricted. This suggests that the circumferential strain can acutely adapt, whereas radial and longitudinal strain has less potential for adaptation.

Since circumferential strain is thought to contribute more to the LVEF than longitudinal strain [33], this could explain why patients with acute myocarditis often have a preserved LVEF despite high myocardial damage, represented by a substantial increase in cardiac biomarkers, ECG changes, and LGE.

LV end-diastolic volume decreased significantly from baseline to follow-up in our study. Importantly, LV dilatation has an influence on strain values due to the change in the fibre course [34, 35]. However, as this is a minor change and its effects should be globally noticeable, we do not assume that this is a source of error in our study.

Our study has several limitations, foremost the retrospective design. While accurate diagnosis of myocarditis remains a challenge, the gold standard of endomyocardial biopsy (EMB) was not performed in this study. However, EMB also has its limitations, mostly resulting from sampling error. Therefore, we applied narrow inclusion criteria and considered only patients with high clinical suspicion of acute myocarditis, according to the ESC Guidelines and positive cardiac MRI criteria for myocardial inflammation, renowned for improving diagnostic accuracy [10, 36]. To minimise confounding, every patient with a relevant comorbidity, which could have an impact on myocardial function, was excluded. Narrow inclusion criteria resulted in a small, but precisely defined population, which conversely might not be generalisable to other myocarditis patient, in particular those without LGE. Moreover, to minimise technical errors we included only patients scanned on the same MRI machine and with identical protocol parameters. Finally, visual qualitative assessment of LGE and segmental strain analysis were performed using the widely used 16-segment model of the AHA. However, due to the focal nature of the disease, taking into account only a single representative basal, midventricular, and apical short axis slice for segmental radial and circumferential strain analysis, as well as a single long axis slice (2CH, 3CH, 4CH) for segmental longitudinal strain analysis might result in a sampling error. The effect of edema as another marker of myocardial inflammation on myocardial function has not been studied, however might have an impact on our results as well.

## Conclusion

This study provides insights into the potential mechanisms of myocardial dysfunction and its compensatory mechanisms over the course of acute myocarditis with preserved ejection fraction derived from segmental feature tracking strain analysis. Our results indicate that compensatory mechanisms are highest for circumferential segmental strain parameters and lowest for longitudinal segmental strain parameters. LGE+ segments show an increase in contractility, indicating recovery. LGE− segments show a decrease in contractility, indicating normalisation after a hypercontractile state or an impairment of an initially normal contracting segment. These effects seem to counterbalance each other, resulting in unchanged global function parameters (LVEF and global strain parameters), which suggests that these parameters might be of limited use in monitoring functional recovery in these patients. Future studies with a larger number of patients and a longer follow-up period are required to further improve our understanding of the underlying pathophysiological process, as well as assessing the long-term effects of the disease on myocardial function.

## Abbreviations

AHA: American Heart Association
BMI: Body mass index
BSA: Body surface area
b-SSFP: Balanced steady-state free precession
CH: Chamber
CI: Confidence interval
ECG: Electrocardiogram
EF: Ejection fraction
ESC: European Society of Cardiology
FWHM: Full width half maximum
kg: Kilogram
LGE: Late gadolinium enhancement
LV: Left ventricle
mm^3^: Cubic millimeter
mmol: Millimole
MRI: Magnetic resonance imaging
ms: Milliseconds
PCS: Peak circumferential strain
PLS: Peak longitudinal strain
PRS: Peak radial strain
RV: Right ventricle
SCMR: Society of Cardiovascular Magnetic Resonance
SD: Standard deviation
SMD: Standardised mean difference
