# Correction: Impact of myocardial injury on regional left ventricular function in the course of acute myocarditis with preserved ejection fraction: insights from segmental feature tracking strain analysis using cine cardiac MRI

**DOI:** 10.1007/s10554-022-02697-7

**Published:** 2022-08-10

**Authors:** L. Weber, J. M. Sokolska, T. Nadarevic, M. Karolyi, B. Baessler, X. Fischer, M. Sokolski, J. von Spiczak, M. Polacin, I. Matziris, H. Alkadhi, R. Manka

**Affiliations:** 1 Institute of Diagnostic and Interventional Radiology, University Hospital Zurich, University of Zurich, Raemistrasse 100, 8091 Zurich, Switzerland; 2 Department of Radiology, Cantonal Hospital Winterthur, Winterthur, Switzerland; 3 Department of Cardiology, University Heart Center, University Hospital Zurich, University of Zurich, Zurich, Switzerland; 4 Department of Heart Diseases, Wroclaw Medical University, Wroclaw, Poland; 5 Department of Radiology, University Hospital Centre Rijeka, Rijeka, Croatia; 6 Department of Sport, Exercise and Health, University of Basel, Basel, Switzerland; 7 Institute for Biomedical Engineering, University and ETH Zurich, Zurich, Switzerland

## Correction to: The International Journal of Cardiovascular Imaging 10.1007/s10554-022-02601-3

In the original publication of the article, the last author name was misspelt. The correct name is given in this correction and the original article has been corrected.

